# *Legionella* pneumonia: increased risk after COVID-19 lockdown? Italy, May to June 2020

**DOI:** 10.2807/1560-7917.ES.2020.25.30.2001372

**Published:** 2020-07-30

**Authors:** Claudia Palazzolo, Gaetano Maffongelli, Alessandra D’Abramo, Luciana Lepore, Andrea Mariano, Antonella Vulcano, Tommaso Ascoli Bartoli, Nazario Bevilacqua, Maria Letizia Giancola, Enrico Di Rosa, Emanuele Nicastri

**Affiliations:** 1 National Institute for Infectious Diseases ‘Lazzaro Spallanzani’ IRCCS, Rome, Italy; 2 Local Health Office, ASL Roma 1, Rome, Italy

**Keywords:** acute respiratory distress syndrome, SARS-CoV-2, Legionella pneumonia, COVID-19 epidemic

## Abstract

We report a case of *Legionella* pneumonia in a dishwasher of a restaurant in Rome, Italy, just after the end of the lockdown that was in place to control the SARS-CoV-2 epidemic. The case highlights the importance of strict monitoring of water and air systems immediately before reopening business or public sector buildings, and the need to consider *Legionella* infections among the differential diagnosis of respiratory infections after lockdown due to the ongoing COVID-19 pandemic.

In response to the coronavirus disease (COVID-19) epidemic in Italy, the Italian Government, on 9 March 2020, imposed a national quarantine [1] and mandated the temporary closure of non-essential shops and businesses, as well as a ban on recreational events. The quarantine lasted at least until 4 May, after which some restrictions were gradually eased, with certain business activities resuming from 18 May onwards. While these non-pharmaceutical measures helped to contain the spread of COVID-19 [2], buildings unoccupied or under-used during the prolonged lockdown pose a risk, through stagnating water in devices or piping systems, of providing conditions where harmful bacteria, including *Legionella*, can proliferate [3]. Here, we describe a case of *Legionella* pneumonia in a restaurant dishwasher, who occurred in conjunction with the end of the lockdown period in Italy, alerting to this potential issue.

## Case presentation

A man aged in his 40s with no comorbidities was admitted to the emergency department (ED) on 3 June 2020 for fever (≥ 38.0°C), dry cough and headache since 28 May. The patient is employed in Rome as a dishwasher in a restaurant that had been closed until 25 May. He worked in the restaurant from 25 to 29 May 2020. Symptoms appeared 3 days after returning to work.

Combined respiratory, droplet and contact isolation measures were prescribed. Two consecutive nasopharyngeal swabs for the detection of severe acute respiratory syndrome coronavirus 2 (SARS-CoV-2), the causative agent of COVID-19, were negative 24 hours apart.

In the ED, a chest computed tomography (CT) scan revealed unilateral single consolidation with air bronchograms in the lower right lobe with an arterial oxygen partial pressure (PaO_2_)/fractional inspired oxygen (FiO_2_) ratio of 376. Blood tests showed hyponatraemia (125 mEq/L, normal range: 135–145 mEq/L); mild leukocytosis (white blood cell count: 12,700/mm^3^, normal range: 4,000–10,000/mm^3^; neutrophil cell count: 8,920/mm^3^, normal range: 1,800–7,500/mm^3^), and elevated C-reactive protein (9.21 mg/dL, normal range: < 1 mg/dL). On 4 June, *Legionella* urinary antigen, serotype 1 was positive and intravenous levofloxacin (750 mg once daily) was started.

On 5 June, the patient was referred to the Lazzaro Spallanzani National Institute for Infectious Diseases in Rome, Italy.

Nasopharyngeal swab and serology for SARS-CoV-2 were both negative. In addition to the urine antigen test, we performed a culture and a PCR for *Legionella* detection, on sputum. Both the culture and PCR were negative. The patient was discharged in good clinical condition after a 14-day course of levofloxacin.

## Environmental investigation

The *Legionella* pneumonia notification led to the alert of the epidemiological surveillance services at regional and district level. An epidemiological investigation plan was set up and multiple environmental samples were taken at the workplace. In the restaurant and in the kitchen where the case worked, there are no air conditioning systems. The staff toilets (shower, sink faucets, sink water and cold water, and hot and cold water bidets) and kitchen taps were sampled and all resulted negative for *Legionella*.

## Discussion

*Legionella* bacteria are aerobic, Gram-negative, intracellular pathogens, with serotype 1 being the most commonly reported cause of human infections worldwide. *Legionella* infections can present as a mild, self-limiting influenza-like illness called Pontiac fever. Alternatively, they can be an important cause of community-acquired and nosocomial pneumonia, with the pneumonic form of infection known as Legionnaire's disease [4]. Symptoms typically arise 2 to 10 days after exposure to the pathogen [4].

The case of *Legionella* pneumonia described in this report became symptomatic 3 days after starting work as a dishwasher. *Legionella* bacteria are typically transmitted via inhalation aerosols from contaminated water or soil [4]. Reported sources of infection are diverse and include showers, pools, hot tubs, aquariums, fountains, birthing pools, drinking water systems, air conditioning systems and cooling towers, and other water collection systems [4]. The environmental contamination by *Legionella* of water systems can be difficult to eradicate as the organism may continue to survive in dead branches of complex plumbing structures.

Given the plethora of water sources to which a person may be exposed during the incubation period [5], determining the source of waterborne *Legionella* infection can be very challenging. In outbreak situations, the source of *Legionella* is commonly sought in order to attempt to stop these; however, for sporadic or isolated cases the finding of the origin of the causative pathogen is rarely pursued [5]. Few data are available on Legionnaires’ disease incidence linked to occupational setting. A review article on occupational Legionnaires’ disease identified 47 reports about 805 workers with nine fatal cases over a period of 66 years (1949–2015; on average, 12.2 cases/year) [6].

Legionnaires’ disease is possibly more common in late summer and early autumn [4]. The case reported here however occurred in spring during the COVID-19 epidemic in Rome. In the catchment area of the Rome 1 health district (ASL Rome 1), 62 Legionnaires’ disease cases were notified in the first semester of 2019 compared with 16 cases in the first semester of 2020, corresponding to a 74% overall case reduction. Whether this might be related or not, to the quarantine imposed in Italy at the beginning of March 2020 is unclear.

Due to the lockdown, the restaurant, where the case worked, had been previously closed. Potential health risks exist in buildings that have been shut down or operating with reduced occupancy during the COVID–19 pandemic [7]. Indeed, this may allow *Legionella* growth in water pipes and equipment using, or dispersing, water, such as evaporative air-conditioning systems or spa pools/tubs, if they are not managed adequately. In this scenario, the implementation of a suitable flushing regime, or other measures, such as draining the system – especially if weekly flushing cannot be maintained, are needed in order to reduce the risk of bacterial infection. In the context of the still ongoing COVID-19 pandemic, a risk reduction strategy for *Legionella* exposure and infection may avoid further overcrowding of already congested EDs.

In January 2020, SARS-CoV-2 infection was identified as the cause of COVID-19 pneumonia [8]. The occurrence in COVID-19 of non-specific symptoms such as fever, cough and dyspnoea implies the need for an accurate differential diagnosis of other causes of community-acquired pneumonia, such as *Legionella. Legionella* pneumonia can moreover lead to a series of radiological findings, including ground-glass opacities or a tree-in-bud appearance in CT, that are compatible with COVID*-*19 [9].

To conclude, although it was difficult to identify the source of infection for the case reported in this study, the importance of monitoring water and air conditioning systems is evident. Reopening of structures related to the work environment has to be conducted with consideration of the full safety of both workers and end-users, and the likely risks of *Legionella* growth have to be evaluated in advance, with decontamination of the water systems. Finally, ED clinicians need to consider *Legionella* pneumonia among other differential diagnoses after the end of the lockdown due to the COVID-19 pandemic.
